# Patterns and characteristics of cognitive functioning in older patients approaching end stage kidney disease, the COPE-study

**DOI:** 10.1186/s12882-020-01764-2

**Published:** 2020-04-09

**Authors:** Floor J. van Deudekom, Marije H. Kallenberg, Noeleen C. Berkhout-Byrne, Gerard J. Blauw, Henk Boom, Jeroen de Bresser, Mark A. van Buchem, André Gaasbeek, Sebastiaan Hammer, Joep Lagro, Matthias J. P. van Osch, Marie-Noëlle Witjes-Ané, Ton J. Rabelink, Marjolijn van Buren, Simon P. Mooijaart

**Affiliations:** 1 Department of Gerontology and Geriatrics C7-Q, Leiden University Medical Center, PO box 9600, 2300 RC Leiden, The Netherlands; 2 Department of Nephrology, Leiden University Medical Center, Leiden, The Netherlands; 3 Department of Geriatrics, Haaglanden Medical Center, The Hague, the Netherlands; 4 Department of Nephrology, Reinier de Graaf Hospital, Delft, The Netherlands; 5 Department of Radiology, Leiden University Medical Center, Leiden, The Netherlands; 6 Department of Radiology, Haga Hospital, The Hague, The Netherlands; 7 Department of Internal Medicine, Haga Hospital, The Hague, The Netherlands; 8 Department of Nephrology, HAGA Hospital, The Hague, The Netherlands; 9 Institute of Evidence-Based Medicine in Old Age, Leiden, the Netherlands

**Keywords:** End stage renal disease, Older patients, Geriatrics, Cognitive function, Geriatric assessment

## Abstract

**Background:**

The prevalence of impaired cognitive functioning in older patients with end stage kidney disease (ESKD) is high. We aim to describe patterns of memory, executive function or psychomotor speed and to identify nephrologic, geriatric and neuroradiologic characteristics associated with cognitive impairment in older patients approaching ESKD who have not yet started with renal replacement therapy (RRT).

**Methods:**

The COPE-study (Cognitive Decline in Older Patients with ESRD) is a prospective cohort study including 157 participants aged 65 years and older approaching ESKD (eGFR ≤20 ml/min/1.73 m^2^) prior to starting with RRT. In addition to routinely collected clinical parameters related to ESKD, such as vascular disease burden and parameters of metabolic disturbance, patients received a full geriatric assessment, including extensive neuropsychological testing. In a subgroup of patients (*n* = 93) a brain MRI was performed.

**Results:**

The median age was 75.3 years. Compared to the normative data of neuropsychological testing participants memory performance was in the 24th percentile, executive function in the 18th percentile and psychomotor speed in the 20th percentile. Independent associated characteristics of impairment in memory, executive and psychomotor speed were high age, low educational level and low functional status (all *p*-values < 0.003). A history of vascular disease (*p* = 0.007) and more white matter hyperintensities on brain MRI (*p* = 0.013) were associated with a lower psychomotor speed.

**Conclusion:**

Older patients approaching ESKD have a high prevalence of impaired memory, executive function and psychomotor speed. The patterns of cognitive impairment and brain changes on MRI are suggestive of vascular cognitive impairment. These findings could be of potentially added value in the decision-making process concerning patients with ESKD.

## Background

Older patients approaching end stage kidney disease (ESKD) have, compared to younger patients, an increased risk for adverse health outcomes in general [1] and for impaired cognitive functioning [2], with a high prevalence ranging from 30 to 87% in dialysis patients [3, 4]. Cognitive impairment has a major impact on outcomes in (older) patients receiving renal replacement therapy (RRT) [5]. Understanding patterns and associated characteristics of cognitive functioning in pre-dialysis and prior to initiating RRT may guide informed treatment decisions and ultimately minimize the risk for further cognitive decline.

Several pathophysiological mechanisms have been suggested as factor contributing to the high prevalence of impaired cognitive function in patients approaching ESKD such as vascular, neurodegenerative and metabolic processes [6, 7]^,^ [8]. Both the brain and kidney are low resistance end organs, exposed to high blood flow and vulnerable to vascular damage [9]. If vascular damage plays a role in developing kidney disease, this may also affect the cerebral vasculature, leading to structural brain abnormalities and cognitive impairment, predominantly in the executive domains and psychomotor speed [10]. Accumulation of uremic toxins may cause cerebral endothelial dysfunction, and lead to neurodegenerative damage in brain regions that play a dominant role in cognitive domains of attention and speed [11]. Only a few studies report on the systematic assessment of patterns of cognitive functioning and their determinants in older patients approaching ESKD prior to treatment initiation. Importantly information on the actual brain damage observed on brain MRI is scarce [12]. This timepoint is of particular importance, as these cognitive impairments may influence treatment decisions and treatment outcome.

In the COPE study [13] we aimed to describe patterns of memory, executive function or psychomotor speed and to identify nephrologic, geriatric and neuroradiologic characteristics associated with cognitive impairment in older patients approaching ESKD who have not yet started with renal replacement therapy RRT.

## Methods

### Study design

The full design of the COPE study, methods and rationale have been published previously [13]. In brief, the COPE study is a prospective, multicentre cohort study in five hospitals in the Netherlands in patients aged 65 years and older reaching ESRD (estimated glomerular filtration rate (eGFR) ≤ 20 ml/min/1.73 m^2^), and attending the pre-dialysis outpatient between April 2014 and December 2017. As part of routine pre-dialysis nephro-geriatric work-up, a comprehensive geriatric assessment (CGA), physical examination, laboratory investigation, neuropsychological testing and a brain MRI scan (in case there was no contra-indication) were performed. The study protocol was approved by the medical ethics committee (METC) of all participating centres.

### Routine renal care

As part of routine pre-dialysis nephrogeriatric work-up, the following clinical parameters were measured: kidney function, metabolic state (urea, phosphate, calcium) and parameters on vascular status (blood pressure, ankle/arm index). eGFR was estimated glomerular filtration rate using the Modified of Diet in Renal Disease (MDRD) [14] or Chronic Kidney Disease Epidemiology Collaboration (CKD-epi) [15] depending on the method used in the different hospitals. Patients were allocated into two groups, vascular and non-vascular cause of kidney disease according to the ERA-EDTA primary renal diagnosis code as assessed by the treating nephrologist. Vascular disease burden was determined as the cause of kidney disease (vascular versus non-vascular), ankle-brachial index, the presence of diabetes and a history of vascular disease (myocardial infarction and/or cerebral vascular incident (CVA) and/or peripheral vascular disease). We considered serum urea, phosphate and calcium as parameters of metabolic disturbance.

### Geriatric work-up

As part of the nephro-geriatric work-up, all patients underwent a comprehensive geriatric assessment (CGA). For a more detailed description of the tests used in the COPE study, see the previously published study protocol [13]. Briefly, the CGA work-up included the following tests: nutrition was assessed using the Normal Subjective Global Assessment score (SGA) [16] and the Short Nutritional Assessment Questionnaire score (SNAQ) [17]. Frailty was assessed using the Fried Frailty Index (FFI) and a score of ≥3 was considered as frail [18]. Functional dependence was assessed by the Groningen Activity Restriction Scale (GARS), where higher scores are indicative of increased dependence (range 18–72) [19], and The Lawton Instrumental Activity of Daily Living score (IADL), with a score ≥ 11 being described as functional dependency [20]. Furthermore, to assess physical capacity the handgrip strength and 6-m gait speed were measured.

### Neuropsychological testing

Trained geriatric or dialysis nurses administered a standardized neuropsychological test battery. It was designed to assess different domains of cognitive functioning such as global cognition, visuoconstruction, memory, executive function and psychomotor speed. The test battery has been successfully used in several study cohorts over the past 20 years [21–23] and is based on clinical experience, scientific literature and relevance for clinical interference [21]. To test global cognition the Mini Mental State Examination (MMSE) was used, with scores ranging from 0 to 30 points where higher scores are indicative of better cognitive performance [24]. Clock drawing was used to assess visuoconstructive abilities and executive function, with scores ranging from 0 to 14 points and higher scores are indicative of better performance [25, 26]. Memory, was assessed using the 15-Word Verbal Learning Test (WVLT) both immediately (total score after five trials) and delayed recall (total score after 20 min), where higher scores indicatedbetter function [27]. To assess memory reproduction the Visual Attention Test (VAT) was used, where higher scores indicated better function [28]. Executive function was assessed with visual attention and task switching was tested with the Trail Making Test A and B (TMT-A and TMT-B), with lower scores indicating better function [29]. To distinguish between processing speed or cognitive (in) flexibility as an explanation of the test result the score on the TMT-B was corrected for the score on the TMT-A. In addition the Stroop Colour Word Test (SCWT) was used, where lower scores indicated better function [30]^,^ [31]. To distinguish between processing speed and cognitive inhibition as an explanation of the test result the score on the Stroop III (interference card) was corrected for the score on the Stroop II (colour naming card). To assess psychomotor speed the Letter Digit Substitution Test (LDST), the Stroop II and the TMT-A was used. For the LDST the number of correct substitutions made in 60 s was used, with higher scores indicating better function [32].

### Normative data of neuropsychological testing

To compare the cognitive test results of the current study with a general population, Dutch normative data for neuropsychological tests corrected for age, gender and educational level were used this to interpret the cognitive status independent of age, gender and level of education [33]. These normative data are frequently used for clinical ratings in daily practice and were available for the 15-WVLT, TMT-A, TMT-B and the SCWT. The norms were based on between 300 and 1000 healthy participants aged 14–90 years.

### MRI of the brain

As part of routine nephro-geriatric work-up a brain MRI was performed in all patients without a contra-indication for MRI. Contraindications for not performing a MRI were: having a pacemaker, having a metallic foreign body (metal sliver) in their eye, being claustrophobic, or not being able to have access to the MRI table due to backpain or impaired mobility. Brain MRI scans were acquired on a Philips Ingenia 3 T scanners at the LUMC (Philips Medical Systems, Best, The Netherlands) according to a standardized scanning protocol. The scanning protocol included T1-weighted images (repetition time (TR) = 8.2 ms; echo time (TE) = 4.5 ms; flip angle 8°, voxel size 1x1x1mm^3^), fluid-attenuated inversion recovery (FLAIR) images (TR = 4800 ms; TE = 313 ms; inversion time (TI) = 1650 ms; voxel size 1.11 × 1.11 × 1.11mm^3^) and susceptibility-weighted imaging (TR = 45 ms; TE 31 ms; flip angle 13°; voxel size 0.8 × 0.8 × 1.6mm^3^). The brain MRI scans were scored for markers of small vessel disease (white matter hyperintensities) and lacunes of presumed vascular origin and microbleeds) according to the STRIVE criteria [34]. White matter hyperintensities were assessed by the Scheltens scale [35].

### Statistical methods

Baseline characteristics are presented as mean with standard deviation (SD) in case of normal distribution, median with interquartile range (IQR) in case of skewed distribution or as number (n) with percentages (%). Mean functioning on the different cognitive domains (memory, executive function and psychomotor speed) are presented as percentiles (mean with IQR), according to the *normative data neuropsychological testing* (see above)*.* To assess associated characteristics of cognitive functioning in different domains, different cognitive tests are stratified in tertiles and mean scores of the different associated characteristics are calculated over the tertiles of cognitive functioning, presented as mean (standard error (SE)). Crude and adjusted *p*-values were calculated with univariable and multivariable linear regression models, respectively, with the continuous score of cognitive performance as dependent variable. In multivariable models we adjusted for age, gender and educational level, to ensure a balanced comparison between the tertiles. The MRI abnormalities were also assessed as determinant of cognitive function. The p-values are presented crude and adjusted (again for age, gender and educational level). All analyses were carried out using SPSS (IBM version 23; IBM Corp., Armonk, New York, USA).

## Results

Table 1 shows the baseline characteristics of the study population. The study population consisted of 157 participants with a median age of 75 years and 103 (66%) participants were male. At study enrolment, the mean eGFR was 16.2 ml/min/1.73m^2^ (standard deviation (SD) 4.4) and over the past 3 years the mean decline in eGFR was 9.1 ml/min/1.73m^2^ (SD 8.0). In 99 (63%) patients a vascular cause, mainly hypertension or diabetes mellitus, was the cause of their primary kidney disease. Almost half of the participants (*n* = 74; 47%) had a history of vascular disease. According to the Fried Frailty Index (FFI) 37 (25%) patients were frail. Functional dependence, as measured by the Instrumental Activities of Daily Living (IADL) with a score of ≥11, was found in 8 (5%) of the patients.

**Table 1:** Baseline characteristics of the included study population

Patient characteristics	
Total	157
Age, median (IQR)	75.3 (70.8–80.8)
Male gender, n (%)	103 (65.6)
Caucasian origin, n (%)	138 (89.0)
Married/living together, n (%)	94 (61.4)
Higher Educational level, n (%)	48 (30.6)
Current smoking	23 (15.0)
Alcohol consumption	77 (50.3)
**Disease specific**	
eGFR at study enrolment, mean (SD)	16.2 (4.4)
Δ eGFR (ml/min), mean (SD)^a^	9.1 (8.0)
Primairy kidney disease	
Non-vascular cause, n (%)	56 (35.7)
Vascular cause, n (%)	99 (63.1)
Diabetes mellitus, n (%)	63 (40.1)
(history of) malignancy, n (%)	47 (29.9)
History of vascular disease, (n%)	74 (47.4)
Ankle-brachial index (right), mean (SD)	0.96 (0.23)
**Medication use**	
Polypharmacy (the use of ≥5 medications), n (%)	139 (89.7)
Glucose lowering medication, n (%)	54 (34.4)
Antihypertensive medication, n (%)	145 (92.4)
Diuretics, n (%)	94 (60.3)
Cholesterol lowering drugs, n (%)	112 (71.3)
Vitamin D supplement, n (%)	131 (83.4)
**Nutrition status**	
Normal Subjective Global Assessment (SGA) score	42 (49.4)
SNAQ score	
Malnourished	8 (10.7)
Risk for malnutrition	9 (12.0)
BMI, median (IQR)	27.4 (24.6–30.9)
Special diet, n (%)	127 (83.0)
**Geriatric assessment**	
Frail according to FFI, n (%)	37 (24.5)
Functional dependence by GARS-score, mean (IQR)	26 (20.0–35.0)
Dependent in IADL function, n (%)	8 (5.0)
Handgrip strength (kg), mean (SD)	
Females	17.2 (6.3)
Males	29.4 (8.1)
Walking speed, mean (SD) (m/s)	1.13 (0.98)

^a^Δ eGFR = difference between eGFR 3 years before and at study enrolment. Abbreviations: *IQR* Interquartile range, *eGFR* Estimated glomerular filtration rate, *SNAQ* Short Nutritional Assessment Questionnaire, *BMI* Body mass index, *FFI* Fried Frailty Index, *GARS-score* Groningen Activity Restriction Score, *IADL* Instrumental Activities of Daily Living. Data complete for; race (*n* = 155), level of education (*n* = 153), marital status (n = 153), smoking and alcohol consumption (*n* = 153), eGFR (*n* = 151), primary kidney disease unknow *n* = 2, polypharmacy (*n* = 155), diet (*n* = 153), SGA-score (*n* = 85), SNAQ = score (*n* = 75), Fried Frailty Index (*n* = 141), Handgrip strength (*n* = 152), walking speed (*n* = 145)

Performance on the global cognitive function and different cognitive domains are reported in the Supplemental Table 1. The population had a median Mini-Mental State Examination (MMSE) of 28 out of 30 points (IQR 27–29). Mean functioning on the memory test (15-Word Verbal Learning Test (15-WVLT)) was in the 24th percentile (IQR 10–54) with a mean score of 31.2 words remembered (SD 9.9). The mean functioning on the executive function (Trail Making Test B (TMT-B)) was in the 18th percentile (IQR 3–54) with a mean score 177.4 s (SD 79.5). The mean functioning on psychomotor speed (Letter Digit Substitution Test (LDST) was in the 20th percentile (IQR 10–50) with a mean score of 21.7 correct substitutions (SD 6.9).

In Tables 2 and 3 and in Supplemental Table 2 we report the associated characteristics of three different cognitive domains, namely memory, executive function and psychomotor speed, respectively. In all three cognitive domains, as expected, older age and lower level of education were significantly associated with cognitive impairment (all *p*-values ≤0.007). For example, the patients who performed in the worst tertile in memory function, compared to the best tertile, were on average 5 years older (*p* < 0.001) and more often received a lower educational level (for memory function: 20% versus 33%, *p* = 0.001).

**Table 2:** Associated characteristics of memory function

	Memory function			***P***-value	
	Best tertile ***N*** = 51	Middle tertile ***N*** = 54	Worst tertile ***N*** = 50	crude	adjusted
Age, mean (SE)	73.8 (0.9)	75.8 (0.9)	78.7 (0.9)	< 0.001	< 0.001*
Gender, n (%)				0.032	0.003*
Female	19 (37.3%)	21 (38.9%)	13 (26%)		
Male	32 (63.7%)	33 (61.1%)	37 (74%)		
Higher educational level, n (%)	17 (33.3%)	20 (37.0%)	10 (20.0%)	0.003	0.001*
eGFR, mean (SE)	16.4 (0.7)	16.1 (0.6)	16.2 (0.6)	0.922	0.664
ΔeGFR, mean (SE)	10.1 (1.7)	8.3 (1.0)	9.1 (1.0)	0.598	0.779
Urea, mean (SE)	20.4 (0.8)	20.9 (0.9)	21.7 (0.8)	0.904	0.582
Phosphate, mean (SE)	1.3 (0.04)	1.3 (0.03)	1.3 (0.04)	0.258	0.527
Calcium, mean (SE)	2.3 (0.02)	2.4 (0.02)	2.3 (0.02)	0.401	0.547
Vascular vs non-vascular cause, n (%)				0.946	0.884
Vascular	28 (56.0%)	39 (72.2%)	31 (63.2%)		
Non-vascular	22 (44.0%)	15 (27.7%)	18 (36.7%)		
Ankle-Brachial index (right), mean (SE)	0.98 (0.03)	0.90 (0.04)	0.99 (0.04)	0.526	0.572
Presence of diabetes, n (%)	18 (35.3%)	24 (44.4%)	21 (42.0%)	0.195	0.286
History of vascular disease, n (%)	19 (37.3%)	26 (48.1%)	28 (56%)	0.004	0.163
Polypharmacy (≥5), n (%)	43 (84.2%)	51 (94.4)	44 (88%)	0.622	0.512
Fried Frailty Index, mean (SE)	1.3 (0.2)	1.6 (0.2)	1.9 (0.2)	0.055	0.082
IADL, mean (SE)	2.0 (0.4)	3.2 (0.5)	4.6 (0.6)	< 0.001	0.003
Walking speed, mean (SE)	1.2 (0.05)	1.0 (0.04)	1.2 (0.25)	0.795	0.545
Handgrip strength, mean (SE)	25.5 (1.4)	24.4 (1.3)	26.1 (1.4)	0.527	0.529

Memory tested by the 15-WVLT. Tertiles of the 15-WVLT: best tertile mean 42.6 (SD 6.3) *n* = 51; middle tertile mean 29.7 (SD 2.8) *n* = 54; worst tertile mean 21 (SD 3.9) *n* = 50 Δ EGFR available for *n* = 41, *n* = 48, *n* = 39. Ankle-Brachial index available for *n* = 35, *n* = 37, n = 39. Walking speed available for *n* = 46, *n* = 50, *n* = 47. Model I: linear regression including correction for age, gender and educational level. *In model I age is only adjusted for gender and educational level; gender is only adjusted for age and educational level; educational level is only adjusted for age and gender

**Table 3:** Associated characteristics of executive function

	Executive function			***P***-value	
	Best tertile ***N*** = 51	Middle tertile ***N*** = 52	Worst tertile ***N*** = 52	crude	model I
Age, mean (SE)	72.9 (0.8)	76.3 (0.9)	78.9 (0.9)	< 0.001	< 0.001*
Gender, n (%)				0.418	0.858*
Female	18 (35.3%)	14 (26.9%)	22 (42.3%)		
Male	33 (64.7%)	38 (73.1%)	30 (57.7%)		
Higher educational level, n (%)	20 (39.2%)	16 (30.8%)	11 (21.2%)	0.003	0.007*
eGFR, mean (SE)	15.6 (0.6)	16.5 (0.6)	16.5 (0.7)	0.246	0.177
ΔeGFR, mean (SE)	10.3 (1.5)	8.0 (1.1)	8.9 (1.1)	0.567	0.962
Urea, mean (SE)	21.1 (0.8)	21.9 (0.9)	19.7 (0.8)	0.100	0.053
Phosphate, mean (SE)	1.4 (0.04)	1.3 (0.03)	1.2 (0.04)	0.064	0.160
Calcium, mean (SE)	2.4 (0.02)	2.3 (0.02)	2.4 (0.02)	0.425	0.299
Vascular vs non-vascular cause, n (%)				0.574	0.566
Vascular	32 (64.0%)	35 (67.3%)	30 (58.8%)		
Non-vascular	18 (36.0%)	17 (32.7%)	21 (41.2%)		
Ankle-Brachial index (right), mean (SE)	0.99 (0.03)	0.89 (0.04)	1.02 (0.04)	0.500	0.303
Presence of diabetes, n (%)	21 (41.2%)	17 (32.7%)	25 (48.0%)	0.199	0.157
History of vascular disease, n (%)	16 (31.4%)	28 (53.8%)	28 (53.8%)	0.012	0.089
Polypharmacy (≥5), n (%)	44 (88.0%)	47 (90.4%)	46 (90.2%)	0.899	0.696
Fried Frailty Index, mean (SE)	1.0 (0.2)	1.7 (0.2)	2.1 (0.2)	< 0.001	0.001
IADL, mean (SE)	1.6 (0.3)	2.7 (0.4)	5.0 (0.6)	< 0.001	< 0.001
Walking speed, mean (SE)	1.2 (0.05)	1.3 (0.2))	0.9 (0.04)	0.089	0.101
Handgrip strength, mean (SE)	27.5 (1.5)	25.9 (1.3)	22.6 (1.2)	0.003	0.020

Executive function assessed by the TMT-B. Tertiles of the TMT-B: best tertile mean 99.5 (SD 21.8) *n* = 51; middle tertile mean 162.8 (SD 21.3) *n* = 52; worst tertile mean 262.2 (SD 37.1) *n* = 52. Δ EGFR available for *n* = 42, *n* = 43, *n* = 43. Ankle-Brachial index available for *n* = 38, *n* = 42, *n* = 31. Walking speed available for *n* = 51, *n* = 47, *n* = 46. Model I: linear regression including adjustment for age, gender and educational level. *In model I age is only adjusted for gender and educational level; gender is only adjusted for age and educational level; educational level is only adjusted for age and gender

Table 2 shows the associated characteristics of the memory domain. After adjusting for age, gender and educational level a higher level of functional dependence (IADL-score) was significantly associated with a more impaired memory function (*p* = 0.003). Patients who performed in the worst tertile of memory function were more functionally dependent compared to the patients who performed in the best tertile (mean IADL-score of 4.6 (SE 0.6) versus a mean IADL-score 2.0 (SE 0.4); *p* < 0.003). Having a history of vascular disease was associated with a more impaired memory function, although the association lost statistical significance after adjustment for age, gender and educational level. Parameters of metabolic disturbance were not associated with an impaired memory function.

Table 3 presents the associated characteristics of the cognitive domain of executive function. After adjusting for age, gender and educational level, a higher level of functional dependence (*p* < 0.001), the presence of frailty (*p* = 0.001) and a lower handgrip strength (*p* = 0.020) were significantly associated with a more impaired executive functioning. For example, in the tertile with the worst executive function, the presence of frailty was higher compared to the best tertile (mean Fried Frailty Index of 2.1 (SE 0.2) versus a mean Fried Frailty Index 1.0 (SE 0.2); *p* = 0.001). Having a history of vascular disease associated with an impaired executive function, although the association lost statistical significance after adjustment for age, gender and educational level. Parameters of metabolic disturbance were not associated with an impaired executive function.

Supplemental Table 2 presents the associated characteristics on the cognitive domain of psychomotor speed. After adjusting for age, gender and educational level, a higher presence of frailty (*p* = 0.001), a higher level of functional dependence (*p* < 0.001) and a lower handgrip strength (*p* = 0.026) were significantly associated with impaired performance on psychomotor speed. For example, the patients who performed in the worst tertile of psychomotor speed had a lower handgrip strength compared to the patients who performed in the best tertile (mean handgrip strength of 24.9 (SE 1.3) versus a mean handgrip strength 26.8 (SE 1.4); *p* = 0.026). After adjusting for age, gender and educational level, having a history of vascular disease was associated with an impaired performance on psychomotor speed (*p* = 0.007). Again, parameters of metabolic disturbance were not associated with an impaired performance psychomotor speed.

The cerebrovascular MRI features in a subpopulation (*n* = 93) are presented in Supplemental Table 3. When comparing the patients with and without a MRI performed, we see that the patients without a MRI are older, more frail, more functional dependent and a higher history of vascular disease, although not statistically significant possibly due to small sample size. The mean Scheltens score of the white matter hyperintensities was 15.8 (SD 7.6). Lobar microbleeds were present in 37 (40%) of the included participants and 19 (20%) participants had non-lobar microbleeds. Lacunes of presumed vascular origin were present in 44 (48%) participants. Table 4 shows which brain MRI abnormalities are associated characteristics of the different neuropsychological domains memory, executive function and psychomotor speed. When adjusting for age, gender and educational level, only a higher burden of white matter hyperintensities was significantly associated with worse psychomotor speed. Patients who performed in the worst tertile of psychomotor speed on average had more white matter hyperintensities compared to patients who performed in the best tertile (mean white matter hyperintensities of 18.6 (SE 1.6) versus a mean white matter hyperintensities 14.6 (SE 1.2); *p* = 0.013). A trend was observed for the association between a higher burden of white matter hyperintensities and lower executive function scores (*p* = 0.054).

**Table 4:** Association between brain MRI features with domains of cognitive function

MRI features	Best tertile	Middle tertile	Worst tertile	***P***-value (crude)	***P***-value (adjusted)^**¥**^
**Memory**					
Presence of microbleeds, n (%)					
Lobar	12 (38.7%)	16 (50%)	9 (31.0%)	0.548	0.287
Non-lobar	9 (29%)	4(12.5%)	6 (20.7%)	0.209	0.048
Presence of lacunes ^a^, n (%)	12 (38.7%)	16 (50%)	15 (51.7%)	0.279	0.635
Total white matter hyperintensities, mean (SE)	14.0 (1.2)	14.9 (1.2)	18.6 (1.7)	0.058	0.096
**Executive function**					
Presence of microbleeds, n (%)					
Lobar	13 (43.3%)	11 (35.5%)	11 (36.7%)	0.821	0.683
Non-lobar	3 (10%)	8 (25.8%)	8 (26.7%)	0.229	0.744
Presence of lacunes^a^, n (%)	14 (46.7%)	14 (46.2%)	14 (46.7%)	0.945	0.635
Total white matter hyperintensities, mean (SE)	13.2 (1.0)	16.0 (1.4)	17.4 (1.6)	0.046	0.054
**Psychomotor speed**					
Presence of microbleeds, n (%)					
Lobar	12 (40%)	12 (38.7%)	13 (46.6%)	0.633	0.871
Non-lobar	5 (16.7%)	7 (22.6%)	7 (21.9%)	0.445	0.993
Presence of lacunes^a^, n (%)	16 (53.3%)	12 (38.7%)	16 (50%)	0.455	0.139
Total white matter hyperintensities, mean (SE)	14.5 (1.2)	14.2 (0.99)	18.6 (1.6)	0.009	0.013

Memory function tested with the 15-WVLT: best tertile mean 43.0 (SD 5.7) *n* = 31; middle tertile mean 31.0 (SD 2.9) *n* = 32; worst tertile mean 21.2 (SD 4.4) *n* = 29. Executive function assessed by the TMT-B: best tertile mean 89.9 (SD 16.3) *n* = 30; middle tertile mean 142.8 (SD 17.7) *n* = 32; worst tertile mean 248.8 (SD 47.2) *n* = 30. Psychomotor speed tested by LDST: best tertile mean 30.1 (SD3 3.1) *n* = 30; middle tertile mean 23.0 (SD 1.9) n-31; worst tertile mean 15.2 (SD 4.0) *n* = 32. ^**¥**^linear regression analysis and adjusted for age, gender and educational level. ^a^Both gliotic and hemorrhagic parenchymal defects in the supratentorial white matter, the brain stem and basal ganglia

## Discussion

The main findings of the present study were twofold. Firstly, impaired cognitive function, in the domains of memory, executive function and psychomotor speed was highly prevalent in patients approaching ESKD not yet started with RRT. Secondly, associated characteristics of a worse cognitive function in the domains memory, executive and psychomotor speed were older age, lower level education, lower functional status, frailty, higher burden of white matter hyperintensities on MRI and a history of vascular disease. Surprisingly, cognitive function was not influenced by parameters of metabolic disturbance.

In the present study, older patients approaching ESKD performed worse on all cognitive domains tested in comparison to the general population. This is consistent with findings in a study of younger patients attending a pre-dialysis clinic in where impairments in psychomotor efficiency and processing speed were more evident than impairments in the domains of learning efficiency or attention and working memory [36]. Similar to our findings one other study [37] of older patients with chronic kidney disease (*N* = 385 median creatinine clearance of 19 ml/min) reported deficits in all cognitive domains, with the largest deficiencies found in recall, attention and executive function. We observed that associated characteristics of a worse cognitive function in the domains memory, executive and psychomotor speed were older age, lower education, lower functional status, frailty, higher burden of white matter hyperintensities on MRI and a history of vascular disease. In other populations with CKD, age, history of falls, functional status and a history of vascular disease were previously described as determinants associated with impaired cognition [6, 37]. A number of studies have reported the prevalence of geriatric impairments, such as dependency in activities of daily living (ADLs) and cognitive impairment, in younger patients with ESKD [38, 39]. The association between white matter hyperintensities and an impaired cognitive function, particularly impairment in attention, executive function and information processing speed, has also been described in older community dwelling and hospitalised patients [40–42]. In our study, parameters of metabolic disturbance (urea, phosphate, calcium) were not associated with a worse cognitive function. There were conflicting results reported on the association of metabolic determinants and the association with a worse cognitive function [11, 43]. In summary, the patterns and associated characteristics of cognitive impairment and the neuroradiological findings in our study population are in line with the previous limited literature.

There are several possible pathophysiological mechanisms that could explain the patterns and associated characteristics of cognitive impairment and the neuroradiological findings in older patients with ESKD described in our study. Firstly, it is possible that ESKD and cerebral vascular damage, are endpoints of the same pathophysiological pathway. Both the brain and kidney share similar vascular anatomy, as low resistance end organs exposed to high volume blood flow into their small vessels, and both have an auto-regulatory system. Because of this unique system, small vessels in kidney and brain, both afferent arterioles and deep perforating arterioles, are particularly prone to be injured by systemic hypertension and other vascular disease [44] as well as by damage due to endothelial dysfunction. Small vessel disease can affect both the kidney and the brain and white matter hyperintensities are considered as a neuroradiological marker for small vessel disease. This could explain the correlation between an impaired renal function and MRI markers of cerebral small vessel disease found in earlier studies [45]. However, extensive research on brain, perfusion and cardiac structure in older ESKD patients is scarce. Second, the high burden of vascular and metabolic morbidity in patients with ESKD lead to an increased biological age, resulting in different phenotypes such as premature vascular aging, muscle wasting, bone disease, cognitive dysfunction and frailty [39]. Taken together, the patterns of cognition and neuroradiological imaging are suggestive of vascular cognitive impairment in older patients with ESKD. Further research is needed to unravel the exact underlying pathophysiological mechanism.

Our results could have some clinical implications. When patients approach ESKD several treatment options, such as RRT including dialysis or transplantation or conservative treatment, are considered. When making treatment decisions, it is important to have insight into the cognitive function of the patient for several reasons. Firstly, cognitive impairment is independently associated with increased mortality, including in patients on RRT [4, 46]. Secondly, patients with cognitive impairment have in general a higher risk for adverse health outcomes such as delirium. Last but not least, it is known that an impaired cognitive functioning can affect decision-making capacity [47] and therefore awareness of cognitive, functional or psycho-social impairment, prior to making a decision on RRT, is of the utmost importance. Information from the geriatric assessment can be taken to the discussions on treatment choice with the patient and family enhancing tailored treatment options for each individual patient.

There were several limitations to the current study. The study is integrated into routine clinical care and possibly has some patient selection bias. For example, the MRI brain is only performed in 60% of the participants, possibly causing some bias. Since the patients not having a MRI where older, more frail, more functional dependent and had a higher history of vascular disease the results described in our manuscript are probably underestimated. It could also be that the patients in worse condition were less likely to participate, which could result in an underestimation of the observed prevalence of cognitive impairment. The study has a relatively small group, which could cause a lack of power. The present analysis reported the cross-sectional association between several associated characteristics and cognition as a consequence that a causal association could not be established. Because of the fact there was not one primary analyses, and this was a post-hoc analysis, we did not perform a formal power calculation. We were however confident that our study provides sensible power to detect clinically relevant findings, because we did find all the known associated characteristics of cognitive function to be statistically significantly associated with cognition in all domains: higher age, higher education level, and higher disabilities in instrumental activities of daily living. Despite these limitations to our knowledge this was the first study in which cognitive function is described so extensively in combination with brain MRI’s in an older population approaching ESKD. The patients included in this study all had an eGFR < 20 ml/min/1.73m^2^ and were not on RRT a study population that has previously only received limited scientific attention. Furthermore, this study focused exclusively on older patients (included median age of 75.3 (IQR 70.8–80.8)), a group whom very often do not participate in clinical trials due to exclusion criteria [48, 49]. With the limited exclusion criteria applied in the COPE-study, the included study population reflected the patients in daily clinical practice.

## Conclusion

Older patients approaching ESKD have a high prevalence of impaired memory, executive function and psychomotor speed. The patterns of cognitive impairment and brain changes on MRI are suggestive of vascular cognitive impairment. These findings could be of potentially added value in the decision-making process concerning patients with ESKD.

## Supplementary information

**Additional file 1: Supplemental Table S1.** Performance on the different cognitive domains.

**Additional file 2: Supplemental Table S2.** Associated characteristics of psychomotor speed.

**Additional file 3: Supplemental Table S3.** Cerebrovascular MRI features in the study population.

## Data Availability

The datasets generated and/or analyzed during the current study are not publicly available, but are available from the corresponding author on reasonable request.

## Abbreviations

CGA: Comprehensive geriatric assessment
CKD-epi: Chronic Kidney Disease Epidemiology Collaboration
COPE-study: Cognitive functioning in Older Patients reaching End stage renal disease
CVA: Cerebral vascular accident
eGFR: Estimated glomerular filtration rate
ESKD: End stage kidney disease
FFI: Fried Frailty Index
FLAIR: Fluid-attenuated inversion recovery
GARS: Groningen Activity Restriction Scale
MDRD: Modified of Diet in Renal Disease
IADL: Lawton Instrumental Activity of Daily Living
IQR: Interquartile range
LDST: Letter Digit Substitution Test
METC: Medical ethics committee
MMSE: Mini Mental State Examination
RRT: Renal replacement therapy
SCWT: Stroop Colour Word Test
SD: Standard deviation
SE: Standard error
SGA: Subjective Global Assessment
SNAQ: Short Nutritional Assessment Questionnaire
TE: Echo time
TMT: Trail Making Test
TI: Inversion time
TR: Repetition time
WVLT: 15-Word Verbal Learning Test
VAT: Visual Attention Test
