# Supplementary material for: Patterns and characteristics of cognitive functioning in older patients approaching end stage kidney disease, the COPE-study
Source: BMC Nephrol. 2020 Apr 9;21:126. doi: 10.1186/s12882-020-01764-2 (PMC7147053; doi:10.1186/s12882-020-01764-2)
Supplement: Supplementary file 1 — Additional file 1: Supplemental Table S1. Performance on the different cognitive domains. [file 12882_2020_1764_MOESM1_ESM.docx]

**Supplemental table 1. Performance on the different cognitive domains**

|  | **Score** | **Percentile***  mean (IQR) |
| --- | --- | --- |
| **Global cognition** |  |  |
| MMSE score (points), median (IQR) | 28 (27-29) |  |
| **Visuoconstruction** |  |  |
| Clock drawing, mean (IQR) | 12 (11-13) |  |
| **Memory** |  |  |
| 15-Word Verbal Learning Test (words remembered) |  |  |
| Immediate recall score, mean (SD) | 31.2 (9.9) | 24 (10-54) |
| Delayed recall score, mean (SD) | 5.8 (3.2) | 22.5 (9.5-58) |
| Visual Association Test (pictures remembered) , median (IQR) | 12 (11-12) | 29.0 (20-29)^×^ |
| **Executive function** |  |  |
| TMT-B (sec), mean (SD)^¥^ | 177.4 (79.5) | 18 (3-54) |
| TMT-B (sec) corrected for TMT-A |  | 27 (12-58) |
| Stroop III (sec), mean (SD) | 172.6 (79.6) | 18 (5-38) |
| Stroop III (sec) corrected for Stroop II (sec), mean (SD) | 88.9 (70.2) | 46 (24-69) |
| **Psychomotor Speed** |  |  |
| LDST (correct in 60 sec), mean (SD) | 21.7 (6.9) | 20 (10-50) |
| TMT-A (sec), mean (SD) | 69.3 (38.5) | 24 (6-56) |
| Stroop II (sec), mean (SD) | 83 (28.9) | 16 (4-31) |

*Corrected for age, gender and educational level.

Abbreviations: IQR= interquartile range, 15-WVLT= 15-Word Verbal Learning Test, TMT= Trail Making Test, Stroop III= Stroop Color Word Test III,

LDST= Letter Digit Substitution Test. Data incomplete for: 15-WVLT (n=155), VAT (n=155), TMT (n=153), STROOP (n=151),

Clock drawing (n=157). ¥: 16 patients did not completed the total test. They have been assigned the maximum number of 300 seconds.

×: 110 patients had the maximum score ending in ≥29th percentile. Score not corrected for age and gender.
