# Supplementary material for: Patterns and characteristics of cognitive functioning in older patients approaching end stage kidney disease, the COPE-study
Source: BMC Nephrol. 2020 Apr 9;21:126. doi: 10.1186/s12882-020-01764-2 (PMC7147053; doi:10.1186/s12882-020-01764-2)
Supplement: Supplementary file 2 — Additional file 2: Supplemental Table S2. Associated characteristics of psychomotor speed. [file 12882_2020_1764_MOESM2_ESM.docx]

**Supplemental table 2. Associated characteristics of psychomotor speed**

|  | **Psychomotor speed** | | |  | **p-value** | | |
| --- | --- | --- | --- | --- | --- | --- | --- |
|  | **Best tertile**  **N=51** | **Middle tertile**  **N=53** | **Worst tertile**  **N=52** |  | **crude** | **model I** |  |
| Age, mean (SE) | 73.9 (0.9) | 75.4 (0.9) | 78.9 (0.9) |  | 0.001 | <0.001* |  |
| Gender, n (%)  Female  Male | 19 (37.3%)  32 (62.7%) | 21 (39.6%)  32 (60.4%) | 14 (26.4%)  39 (73.6%) |  | 0.284 | 0.038* |  |
| Higher educational level, n (%) | 21 (41.2%) | 16 (30.2%) | 11 (20.8%) |  | <0.001 | <0.001* |  |
| eGFR, mean (SE) | 16.8 (0.7) | 15.4 (0.5) | 16.3 (0.6) |  | 0.319 | 0.217 |  |
| ΔeGFR, mean (SE) | 9.3 (1.3) | 10.3 (1.3) | 7.8 (1.1) |  | 0.920 | 0.635 |  |
| Urea, mean (SE) | 20.1 (0.9) | 21.7 (0.9) | 21.2 (0.8) |  | 0.138 | 0.247 |  |
| Phosphate, mean (SE) | 1.3 (0.03) | 1.3 (0.04) | 1.3 (0.03) |  | 0.934 | 0.620 |  |
| Calcium, mean (SE) | 2.4 (0.02) | 2.3 (0.02) | 2.4 (0.02) |  | 0.711 | 0.835 |  |
| Vascular vs non-vascular cause, n (%)  Vascular  Non-vascular | 28 (54.9%)  22 (43.1%) | 35 (66.0%)  18 (34%) | 36 (67.9%)  16 (30.2%) |  | 0.856 | 0.875 |  |
| Ankle-Brachial index (right), mean (SE) | 0.95 (0.03) | 0.96 (0.03) | 0.98 (0.05) |  | 0.927 | 0.732 |  |
| Presence of diabetes, n (%) | 15 (29.4%) | 26 (49.0%) | 22 (41.5%) |  | 0.426 | 0.589 |  |
| History of vascular disease, n (%) | 15 (29.4%) | 24 (45.3%) | 35 (67.3%) |  | <0.001 | 0.007 |  |
| Polypharmacy (≥5), n (%) | 45 (88.2%) | 46 (86.8%) | 48 (90.6%) |  | 0.413 | 0.918 |  |
| Fried Frailty Index, mean (SE) | 1.1 (0.2) | 1.7 (0.2) | 2.0 (0.2) |  | <0.001 | 0.001 |  |
| IADL, mean (SE) | 1.3 (0.3) | 3.1 (0.4) | 5.3 (0.6) |  | <0.001 | <0.001 |  |
| Walking speed, mean (SE) | 1.2 (0.04) | 1.2 (0.2) | 0.9 (0.04) |  | 0.123 | 0.067 |  |
| Handgrip strength, mean (SE) | 26.8 (1.4) | 24.3 (1.3) | 24.9 (1.3) |  | 0.102 | 0.026 |  |

Associated characteristics of psychomotor speed tested nu the LDTS. Tertiles of the LDST: best tertile mean 29.5 (SD 3.2) n=51; middle tertile mean 21.7 (SD 1.8) n=53; worst tertile mean 14.2 (SD 3.7) n=52. Δ EGFR available for n=45, n=43, n=42. Ankle-Brachial index available for n=33, n=41, n=38.

Walking speed available for n=46, n=48, n=51. Model I: linear regression including adjustment for age, gender and educational level. *In model I age is only adjusted for gender and educational level; gender is only adjusted for age and educational level; educational level is only adjusted for age and gender.
