# Supplementary material for: Patterns and characteristics of cognitive functioning in older patients approaching end stage kidney disease, the COPE-study
Source: BMC Nephrol. 2020 Apr 9;21:126. doi: 10.1186/s12882-020-01764-2 (PMC7147053; doi:10.1186/s12882-020-01764-2)
Supplement: Supplementary file 3 — Additional file 3: Supplemental Table S3. Cerebrovascular MRI features in the study population. [file 12882_2020_1764_MOESM3_ESM.docx]

**Supplemental Table 3. Cerebrovascular MRI features in the study population**

| **MRI feature (n=93)** | **Prevalence** |
| --- | --- |
| Presence of microbleeds, n (%) |  |
| Lobar | 37 (39.8%) |
| Non-lobar | 19 (20.4%) |
| Presence of lacunes*, n (%) | 44 (47.3%) |
| Total white matter hyperintensities  (Scheltens score), mean (SD) | 15.8 (7.6) |

*Both gliotic and hemorrhagic parenchymal defects in the supratentorial white matter, the brain stem and basal ganglia.

Data complete for: microbleeds (lobair (n=93), non-lobair and cerebellair (n=92)), lacunes (n=93)
